# Relapsing–remitting clinical course expands the phenotype of Aicardi–Goutières syndrome

**DOI:** 10.1002/acn3.50979

**Published:** 2020-01-10

**Authors:** Jeffrey Lambe, Olwen C. Murphy, Weiyi Mu, Krista Sondergaard Schatz, Kristin W. Barañano, Arun Venkatesan

**Affiliations:** ^1^ Department of Neurology Johns Hopkins University School of Medicine Baltimore Maryland; ^2^ Department of Genetic Medicine Johns Hopkins University School of Medicine Baltimore Maryland

## Abstract

Aicardi–Goutières syndrome (AGS) is a rare and likely underdiagnosed genetic leukoencephalopathy, typically presenting in infancy with encephalopathy and characteristic neuroimaging features, with residual static neurological deficits. We describe a patient who, following an initial presentation at the age of 12 months in keeping with AGS, exhibited a highly atypical relapsing course of neurological symptoms in adulthood with essentially normal neuroimaging. Whole‐exome sequencing confirmed a pathogenic *RNASEH2B* gene variant consistent with AGS. This case highlights the expanding phenotypes associated with AGS and the potential role of whole‐exome sequencing in facilitating an increase in the rate of diagnosis.

## Introduction

Aicardi–Goutières syndrome (AGS) is a rare genetic leukoencephalopathy which typically presents in utero, resembling a congenital infection, or within the first 4 months of life following an uncomplicated pregnancy and normal early development.^1^ It generally manifests as an initial subacute encephalopathy with regression of motor development and progressive microcephaly over several months, followed by clinical stabilization and residual fixed cognitive deficits and spastic‐dystonic tetraplegia.^1^ Neuroimaging characteristically demonstrates cerebral atrophy, intracranial calcifications, and/or demyelinating or delayed myelination patterns in white matter.^1^, ^2^ Pathogenic variants in over seven genes including *ADAR, RNASEH2A, RNASEH2B, RNASEH2C, SAMHD1, TREX1,* and *IFIH1* have been associated with AGS to date, while it is likely that new sequencing technologies will identify further mutations involved in disease pathogenesis in the future. Inheritance is typically autosomal recessive. A subset of AGS patients with pathogenic variants in *ADAR, SAMHD1, IFIH1,* and *RNASEH2B* have been reported to exhibit a more variable phenotype, with later symptom onset, better preservation of motor and cognitive function, and partial or complete absence of classical neuroimaging features.^3^, ^4^, ^5^, ^6^, ^7^, ^8^, ^9^ Herein, we describe a patient whose relapsing clinical course further broadens the phenotype of AGS associated with pathogenic *RNASEH2B* variants.

## Case Report

A 12‐month‐old girl, born of nonconsanguineous parents of European descent and exhibiting normal early development, first presented after developing cyclical fevers, around which time her parents noted that she had started dragging her left leg when walking. Over several days, her symptoms evolved to include bilateral leg weakness and loss of language skills. There was no evidence of microcephaly or stagnation of head circumference growth, with no intracranial calcifications or other abnormalities observed on a computed tomography (CT) scan. Magnetic resonance imaging (MRI) at that time revealed approximately 10 T2 hyperintensities supratentorially (images unavailable), and she was diagnosed with acute disseminated encephalomyelitis (ADEM). There was no further progression of her symptoms, although she did have residual static spasticity in both lower limbs necessitating an L1/L2 dorsal rhizotomy at the age of 10 years and use of bilateral canes when ambulating. Her language skills recovered spontaneously over several months; cognition was normal throughout childhood and adolescence.

From age 23 years onwards, she experienced recurrent discrete episodes of severe spasticity and weakness in her legs and arms, accompanied by dysarthria and dysphagia. She noted several potential triggers preceding the onset of these episodes, including physiological stress, illness, and the beginning of her menstrual cycle. The severity of her spasticity often fluctuated from day to day, with no clear pattern identified. Episodes lasted for several months at a time, requiring multiple hospitalizations. The longest of these episodes necessitated a 5‐month inpatient hospitalization with percutaneous endoscopic transgastric jejunostomy tube placement due to the severity of her dysphagia, with a further month of inpatient rehabiltation. Episodes were typically followed by periods of slow, spontaneous, incomplete improvement. Despite regular engagement with outpatient physiotherapy and rehabilitation services, her mobility declined such that she required a rollator for short distances and a motorized wheelchair for longer distances. Cognition was never affected; she completed a bachelor's degree as her highest level of education. Throughout this period she was thought to have an inflammatory or demyelinating central nervous system (CNS) disorder and several immunotherapy treatments were trialed, including high‐dose corticosteroids, plasmapheresis, intravenous immunoglobulin (IV Ig) and mycophenolate mofetil, with no perceptible therapeutic effect. She experienced mild symptomatic benefit from dalfampridine and nocturnal diazepam.

She first attended our clinic at the age of 24 years. On examination, her mental status was normal with fluent speech and mild spastic dysarthria. Cranial nerve examination, including extraocular movements, was normal. Upper extremity strength was 4+/5 on medical research council (MRC) scoring, with mild spastic tone. Strength was 2/5 hip and knee flexion bilaterally, and 4/5 dorsiflexion bilaterally. There was moderate spasticity in the lower extremities, with 3+ reflexes throughout. Cerebellar examination and sensation were normal throughout. She could stand with assistance and ambulate a short distance with the use of a rollator.

**Figure 1:**
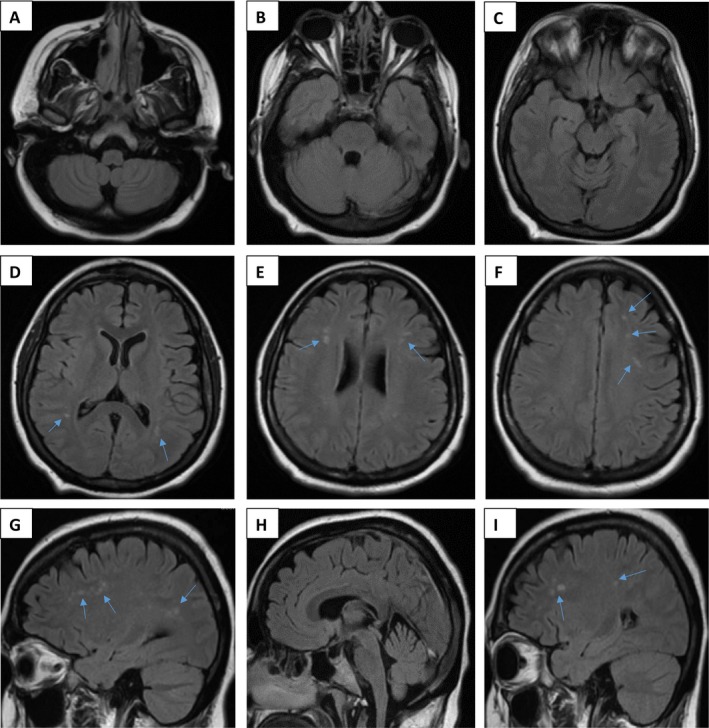
MRI findings. Neuroimaging (T2 fluid‐attenuated inversion recovery [FLAIR]) of the patient performed at 30 years of age (A–F: axial views [A–C: level of medulla oblongata, pons, and midbrain, respectively]; G–I: sagittal views), demonstrating scattered small foci of hyperintensity within the periventricular and subcortical white matter (indicated by arrows).

Cerebrospinal fluid (CSF) analysis was negative for oligoclonal bands and primary CNS infections, with normal cell count, and glucose, protein, lactic acid, very long chain fatty acids and free carnitine within reference ranges. CSF alpha‐interferon levels were not measured. Lower limb electromyogram was unremarkable. MRI brain demonstrated several small subcortical T2 hyperintensities (Fig. 1), unchanged on serial examinations over several years, without enhancement or restricted diffusion. There was no evidence of cerebral atrophy, calcifications, or other intracranial or spinal cord abnormalities on either MRI or CT imaging. Despite a negative family history for neurological or autoimmune disease, genetic testing was pursued given the unusual clinical course and nondiagnostic neuroimaging. Trio whole‐exome sequencing revealed her to be homozygous for a known pathogenic *RNASEH2B* gene variant, c.529G>A (p.A177T), consistent with AGS, with no secondary variants identified. Her parents were each found to be heterozygous carriers.

## Discussion

We report a patient whose initial presentation in infancy resembling hereditary spastic paraplegia with extensive white matter abnormalities and residual neurological deficits was in keeping with the known history of AGS,^3^ as was the presence of the *RNASEH2B* missense mutation c.529G>A.^2^ However, her subsequent clinical course was highly unusual. While most patients experience a “plateau” of neurological symptoms without further progression beyond the initial phase, our patient experienced a highly atypical relapsing course of neurological symptoms commencing in her third decade of life. We note one previously described case of recurrent encephalopathy in an AGS patient with an *ADAR1* mutation.^10^ Although the prominent spasticity experienced by the patient is characteristic of AGS, dysarthria and dysphagia have rarely been described in association with the disorder.^11^, ^12^ Additionally, her speech and swallowing difficulties, suggestive of brainstem dysfunction, do not correlate with her neuroimaging, which is in itself remarkable for the absence of extensive MRI abnormalities relative to her initial ADEM‐like presentation; while radiological improvement following the initial disease phase has been described in isolated cases of AGS,^13^ white matter abnormalities generally remain stable over time.^7^

The natural history of milder presentations of AGS has only been partially delineated. While fewer than 200 cases of c.529G>A *RNASEH2B* mutations have been described, the predicted prevalence of AGS due to biallelic c.529G>A mutations is 1 in 250,000 in the general population and 1 in 120,000 in the European population, suggesting that the condition may be largely underdiagnosed.^3^ The increasing availability of whole‐exome sequencing and subsequent genotype‐first diagnostic approach may facilitate an increase in the recognition of AGS and, in turn, the spectrum of phenotypes associated with the disease.^3^ The identification of an *RNASEH2B* mutation in this patient through whole‐exome sequencing, with no secondary variants identified, confirmed a diagnosis of AGS that had until that point remained elusive despite extensive investigation. Intriguingly, although *RNASEH2B* mutations comprise the majority of mutations in milder disease, they have also been frequently observed in the “typical” phenotype of the disease.^2^ This may suggest epigenetic modifiers or environmental factors altering the effect of gene mutations associated with AGS, thereby modifying disease phenotype. The influence of these as yet unidentified factors on specific aspects of AGS, such as abnormalities in the interferon‐alpha‐mediated immune response which are believed to be central to disease pathogenesis, is an area that requires further research.^6^, ^14^ The significance of potential triggers – such as illness and menstruation, as identified by the patient in this case – and their effect on interferon levels similarly remains to be determined.

While the neurological damage accrued early in the disease course is likely irreversible, the identification of a relapsing–remitting disease course in AGS may present an opportunity for initiation of treatment to attenuate further decline.^15^ Type I interferon upregulation in AGS is not limited to the initial encephalopathic disease stage and may be a lifelong phenomenon. Therapies to block interferon signaling may therefore be of utility in patients exhibiting recurrent symptomatology, and several Janus kinase inhibitors have shown early promise in several type I interferonopathies.^16^, ^17^, ^18^, ^19^ Additionally, patients with mutations in the RNASE H2 complex in particular exhibited reductions in interferon signaling following treatment with a combination of anti‐human immunodeficiency virus‐1 reverse transcriptase inhibitors (abacavir, lamivudine, and zidovudine).^20^ “Broad‐spectrum” immunosuppression (for example, plasmapheresis, corticosteroid, IV Ig, and mycophenolate mofetil therapy as trialed in this case) has failed to demonstrate notable improvement in isolated case reports of AGS.^8^, ^18^, ^21^

This case highlights the clinical and radiological heterogeneity of AGS, a condition that is likely under‐recognized and therefore underdiagnosed. In adult patients with spasticity, episodic neurological symptoms and nonspecific white matter abnormalities, clinicians should thoroughly investigate the past clinical history, looking for more typical AGS features (subacute/acute onset of motor regression, irritability, and recurrent fevers) that could have been missed or overlooked.

## Conflict of Interest

The authors declare no conflicts of interest.

## Author Contribution

JL wrote and revised the manuscript, and acquired and interpreted the data. OCM, WM, KSS, and KWB interpreted the data and revised the manuscript for intellectual content. AV designed and conceptualized the case, revised the manuscript for intellectual content, and took responsibility for the data, accuracy of data analysis and interpretation of the data.

## Ethical Statement

We confirm that we have read the journal's position on issues involved in ethical publication and affirm that this report is consistent with those guidelines.
